# Tracking and forecasting milepost moments of the epidemic in the early-outbreak: framework and applications to the COVID-19

**DOI:** 10.12688/f1000research.23107.2

**Published:** 2020-09-18

**Authors:** Huiwen Wang, Yanwen Zhang, Shan Lu, Shanshan Wang

**Affiliations:** 1 School of Economics and Management, Beihang University, Beijing, China; 2 Beijing Advanced Innovation Center for Big Data and Brain Computing,, Beijing, China; 3 School of Statistics and Mathematics, Central University of Finance and Economics, Beijing, China; 4 Beijing Key Laboratory of Emergence Support Simulation Technologies for City Operations, Beijing, China

**Keywords:** COVID-19, Prediction Method, Epidemic Development Index System

## Abstract

**Background:** The outbreak of the 2019 novel coronavirus (COVID-19) has attracted global attention. In the early stage of the outbreak, the most important question concerns some meaningful milepost moments, including the time when the number of daily confirmed cases decreases, the time when the number of daily confirmed cases becomes smaller than that of the daily removed (recovered and death), and the time when the number of daily confirmed cases and patients treated in hospital, which can be called “active cases”, becomes zero. Unfortunately, it is extremely difficult to make right and precise prediction due to the limited amount of available data at the early stage of the outbreak. To address it, in this paper, we propose a flexible framework incorporating the effectiveness of the government control to forecast the whole process of a new unknown infectious disease in its early-outbreak.

**Methods**: We first establish the iconic indicators to characterize the extent of epidemic spread. Then we develop the tracking and forecasting procedure with mild and reasonable assumptions. Finally we apply it to analyze and evaluate the COVID-19 outbreak using the public available data for mainland China beyond Hubei Province from the China Centers for Disease Control (CDC) during the period of Jan 29th, 2020, to Feb 29th, 2020, which shows the effectiveness of the proposed procedure.

**Results**: Forecasting results indicate that the number of newly confirmed cases will become zero in the mid-early March, and the number of patients treated in the hospital will become zero between mid-March and mid-April in mainland China beyond Hubei Province.

**Conclusions:** The framework proposed in this paper can help people get a general understanding of the epidemic trends in countries where COVID-19 are raging as well as any other outbreaks of new and unknown infectious diseases in the future.

## 1 Introduction

The atypical pneumonia caused by the 2019 novel coronavirus (COVID-19), which is a highly infectious human disease, was first reported in Dec 31st, 2019 in Wuhan, the capital of Hubei Province in China ( WHO *et al.*, 2020). To mitigate the effect of epidemics spreading across China and other countries, Wuhan was temporarily shut-down from Jan 23th, 2020, which has proved to be efficient in the timely stopping the spread of the coronavirus ( Chinazzi *et al.*, 2020). However, due to the “Spring Festival travel rush”, there was still a rising number of confirmed cases in China in the following two months, which has caused great strain on medical resources ( Li *et al.*, 2020).

The questions that draw the most concerns are how COVID-19 will spread, and when it will end. People were always asking when the number of the daily confirmed cases will become smaller than the previous days, and when the daily confirmed cases will become smaller than that of the removed (recovered and death). These are not only of highly important for the general public, but also for government, who plays an important role in controlling the disease within a short period as much as possible. Since the decline of the number of newly confirmed cases and the number of active cases imply the alleviation of epidemic, the emergence of these turning points convey useful information for decision making on medical resources allocation and isolation policies in the post-stage of the epidemic.

Meanwhile, it is also important to predict when will the number of daily confirmed cases become “zero”, as well as when the number of active cases will be “zero”. The latter indicates the end of the epidemic. These two “zero points” can also help the government to consider loosening population migration restriction in cities. Additionally, authorities in economic departments can use the forecasting results to assess the impact of the epidemic on the economy in advance, and plan for the restoration of normal production and living order.

There have been various publications on COVID-19 from different perspectives, i.e., the origin of COVID-19, the clinical features as well as epidemic transmission characteristics. Specifically, for the origin of the virus, Fan *et al.* (2019) and Luk *et al.* (2019) pointed out that COVID-19 is an infectious disease caused by a virus closely related to SARS-CoV, while others believed that the COVID-19 virus was originally derived from wild animals ( Benvenuto *et al.*, 2020; Huang *et al.*, 2020). For the epidemic transmission characteristics, Holshue *et al.* (2020) and Hui *et al.* (2020) found that the virus can be transmitted from person to person and that it has a high interpersonal transmission rate. Zhao *et al.* (2020) investigated the preliminary estimation of the basic reproduction number *R*_0_, which ranged from 2.24(95%CI : 1.96 − 2.55) to 3.58(95%CI : 2.89 − 4.39) in the early outbreak, while Prasse *et al.* (2020) estimated it around 2.2, Tang *et al.* (2020) applied likelihood-based and model-based methods to the analysis of early reported cases, and the results showed that *R*_0_ could be is as high as 6.47. Zhou *et al.* (2020) used the SEIR model and stated that the range of *R*_0_ of COVID-19 is 2.8–3.3, indicating that the early pathogenic transmission capacity of COVID-19 is close to or slightly higher than SARS. Other studies related to *R*_0_ are Anastassopoulou *et al.* (2020); Zhang *et al.* (2020) and referenced therein. Unfortunately, each of these models may result in different estimations of *R*_0_, which may cause any predictions based on *R*_0_ to be unstable.

Recently, a number of publications have been related to trend prediction of the COVID-19 outbreak in China. Zeng *et al.* (2020) proposed a multi-model ordinary differential equation set neural network and model-free methods to predict the interprovincial transmission in mainland China, especially those from Hubei Province, and predicted that COVID-19 in China is likely to decelerate before Feb 18th and to end before April 2020. Chen *et al.* (2020) made prediction based on epidemiological surveys and analyses, which showed that the total number of diagnoses would be 2–3 times that of SARS, and the peak is predicted to be in early or middle February. Yu *et al.* (2020) revised the SIR model based on the characteristics of the COVID-19 epidemic development, and proposed a time-varying parameter-SIR model to study the trend of the number of infected people. Peng *et al.* (2020) used the SEIR method to predict the end of the epidemic in most cities in mainland China. Wu *et al.* (2020) used the Markov chain Monte Carlo method to estimate *R*_0_, and inferred from the SEIR model that the peak COVID in Wuhan would be reached in April, and other cities in China would be delayed by 1 to 2 weeks.

However, there are some obvious shortcomings of forecasting methods based on epidemic models in terms of outbreak prediction. For example, the SEIR model is a mathematical method relying on an assumption of epidemiological parameters for disease progression, which are absent for a novel pathogen. For instance, the basic infection number *R*_0_, the daily recovery rate, the characteristics of the disease itself (such as the infection rate and the conversion rate of the latent to the infected), the daily exposure rate of the latent and infected, and their initial population infection status (total population, infected, the initial value of the latent, the susceptible, the healer, etc.) and many other key parameters need to be set. For infectious diseases that have already appeared in the past, or those who have a large amount of data, it is not difficult to obtain these parameters. However, for unknown, sudden and early infectious diseases, obtaining these parameters is full of difficulties, which leads to a great uncertainty and limitations in the prediction of the epidemic situation using the SEIR model.

Moreover, there exist many challenges for the prediction of a new epidemic situation similar to COVID-19. First, little prior knowledge that can be refered to or analogized for a brand new epidemic; secondly, the existence of government management will make the development of the epidemic completely different from that under free development, thus how to incorporate the influence of government measures into the fitting process of parameters and build a statistical model from this needs to be considered; thirdly, in the early-outbreak the initial data often fluctuates violently and the data quality is low, thus many commonly used parameter estimation methods are not applicable anymore; furthermore, the amount of data in the early stage is too small, making it difficult to directly rely on the inertia of the data to make forward prediction. In summary, in the early stages of a brand new epidemic, how to use some low-quality and small data sets to make basic and relatively accurate forecast judgements for the entire process of the epidemic, is a long-term pain point.

To cope with these challenges, we propose a simple and effective framework incorporating the effectiveness of the government control to forecast the whole process of a new unknown infectious disease in its early-outbreak, from which we emphasis the prediction of meaningful milepost moments. Specifically, we first propose a series of iconic indicators to characterize the extent of epidemic spread, and describe four periods of the whole process corresponding to the four meaningful milepost moments: two turning points and two “zero” points; then we develop the proposed procedure with mild and reasonable assumptions, specfically without relying on an assumption of epidemiological parameters for disease progression. Finally we apply it to analyze and evaluate COVID-19 using publicly available data from mainland China beyond Hubei Province from the China CDC during the period of Jan 29th, 2020, to Feb 29th, 2020, which shows the effectiveness of the proposed procedure.

From the empirical study, we can suggest that the proposed method may cast a flexible framework and perspective for early prediction of a sudden and unknown new infectious disease with effective government control. Specifically, in the early stage of the epidemic when some regular information is initially displayed, the proposed method can be used to predict the process of epidemic development and to judge which stage of development the situation is at, when the peak will be reached, and when the turning point will appear. Moreover, by continuously accumulating data and updating the model during the development of the epidemic, we can also predict when the epidemic will basically end. Finally, the proposed method enjoys great generalizability, which can be used to understand the epidemiological trend of COVID-19 spread in other counties, which will provide useful guidance for fighting against it.

The reminder of this paper is organized as follows. In Section 2, we proposed the main methodology, where we defined the iconic indicators to characterize the extent of epidemic spread in Section 2.1, yielding four periods of the whole process corresponding to the four meaningful milepost moments: two turning points and two “zero” points in Section 2.2, then Section 2.3 presents the proposed procedure with mild and reasonable assumptions. Then we applied the proposed method to the COVID-19 using the public available data in mainland China beyond Hubei Province from the China CDC during the period of Jan 29th, 2020, to Feb 29th, 2020, and describe the trend of the COVID-19 spread in detail in Section 3. Some conclusions and discussions are finally given in Section 4.

## 2 Methods

The data we used are provided by China CDC via public data sources, in which the cumulative confirmed cases up to the given day *t*, the daily confirmed cases at day *t*, the daily recovered ones and the daily deaths at day *t* are included. All the data analysis results are done with **R** software, version 3.6.0 and higher is recommended. The main code for the implementation of the proposed procedure as well as the data and its full description are available from **Github** (See data availability for more detail ( YuanchenZhu2020, 2020).

In order to assess and predict the epidemic, we first define a set of necessary indicators that can reflect the status of disease contagion. We then divide the cycle of the epidemic into four stages, which are divided by the turning points of the proposed indicators. Finally, we propose a computational framework to predict the turning points.

### 2.1 The iconic indicators to characterize a epidemic

It is obvious that the contagion process of an unknown virus in different regions would be diverse with respect to the number of patients and the growth pattern of the epidemic, because of population density, population mobility, public health conditions, as well as disease prevention and control measures. Therefore, we first constructed a set of indicators to monitor the essential laws of the development of the disease.

There are several requirements for the monitoring indicators. Firstly, as the number of patients can vary greatly across regions, the scale of the data should be eliminated so that the analysis methods and results are comparable. Secondly, they should well reflect the general laws and characteristics of the epidemic process as well as accurately and coherently describe the entire process of the epidemic from the beginning to the end. Particularly, they should be able to answer the question of when the turning point of the epidemic would appear. Thirdly, they should be as simple and convenient as possible so that it can be applied with publicly available data. Last but not least, the indicators should have clear meaning and be easily interpreted.

Following the above, we first adopt three basic indicators that are published daily by the provincial and municipal governments of China. That is, for time *t*, the daily confirmed cases *E _t_*, the daily recovered cases *O _t_*, and the daily deaths *D _t_*. Then we define a few monitoring indicators to characterize the epidemic stages, that is the number of active cases *N _t_*, the daily infection rate *K _t_* and the daily removed (the sum of recovered and deaths) rate *I _t_*, which are defined as follows.

- The number of active cases *N _t_* is defined as the cumulative confirmed cases with recovered ones and deaths removed up to *t*, that is
  $$N_{t}=\sum_{i=1}^{t}\left(E_{t}-O_{i}-D_{i}\right).$$
  Note that *N _t_* is essential for epidemic investigation, since it reflects the size of local patients and the pressure on the medical system.
- The daily infection rate *K _t_* is defined as the ratio of the daily confirmed cases at time *t* and the number of active cases at time *t* − 1, i.e.
  $$K_{t}=\frac{E_{t}}{N_{t-1}}.$$
  Obviously, *K _t_* reflects the rate at which patients enter the treatment system. It is influenced by many factors, including the property of the infectious disease, the average immune capacity of the population, population density, climate condition, public health conditions, public health awareness, the awareness of self-prevention of diseases and the efforts of epidemic prevention and control.
- Similarly, the daily removed rate *I _t_* is defined as the ratio of the daily removed cases at time *t* and the number of active cases at time *t* − 1, i.e.
  $$I_{t}=\frac{O_{t}+D_{t}}{N_{t-1}},$$
  where *I _t_* reflects the rate at which patients leave the medical system, that is, the rate at which the pressure on medical resource is eased.

Using the above indicators, we further define *R _t_* as the outbreak status on day *t* as follow:

$$R_{t}=1+K_{t}-I_{t}.$$

Obviously, it holds that

$$N_{t}=N_{t-1}R_{t}=N_{0}\prod_{l=1}^{t}\left(1+K_{l}-I_{l}\right),\;(1)$$

where *N*_0_ denotes the initial number of active cases at the beginning of the outbreak. In particular, when the daily infection rate and removed rate are relatively stable, denoted as *K* and *I* respectively, we have the constant epidemic status index *R* = 1 + *K* − *I*. Then ( 1) can be written as:

$$N_{t}=N_{0}\cdot R^{t}=N_{0}\cdot {\left(1+K-1\right)}^{t},\;(2)$$

which shows that the epidemic situation is in the form of an exponential curve. And the epidemic status indicator *R* can well reflect the rate of expansion or convergence of the population with infectious capacity.

### 2.2 Four stages of an epidemic

In this section, we will describe the whole process of a epidemic under the assumption that the government has implemented effective control measures, which can be divided into four stages, i.e. “outbreak period”, “controlled period”, “mitigation period” and “convergence period” successively. And we will quantify the iconic features for each stage, which corresponds to the two turning points and two “zero” points, respectively.

**Stage 1: Outbreak Period**

In the initial stage of an epidemic outbreak, there is delay of social response due to the limited knowledge of the epidemic, and the power of contagion prevention and control is inevitably not enough. Thus the daily infection rate *K _t_* would be high. At the same time, the recovery process in the initial stage is relatively long, and the number of severe patients is small, leading the daily removed rate *I _t_* to be close to “zero”. Therefore, the outbreak status indicator *R _t_* during this period is usually much larger than 1, that is:

$$K_{t}\gg I_{t},R_{t}=1+K_{t}-I_{t}>1\Rightarrow N_{t}>N_{t-1}.$$

It can be seen that, during the outbreak period, the number of newly diagnosed patients increases sharply, and the number of active cases will increase dramatically correspondingly, which will pose a great burden to medical institutions, especially for hospitals.

As the epidemic exacerbates, if the government begins to intervene through a series of emergency measures, where a disease prevention and control system is quickly established, the daily infection rate *K _t_* will significantly decrease. Usually, the new daily confirmed cases will begin to decline as well. During the epidemic prevention and control process, once the situation improves, we will see the emergence of the first turning point denoted as *T*_1_. Then after the data *T*_1_, the newly diagnosed patients *E _t_* changes from a rapid rise in the outbreak period to a descending channel ( *E _t_* < *E*_*t*−1_). In summary, the emergence of the first turning point *T*_1_ indicates that the disease control measures have begun to work, which implies the end of the “Outbreak Period”.

**Stage 2: Controlled Period**

The emergence of the first turning point is a very positive signal, indicating that the public health management measures have obviously taken effect and the epidemic has entered the “controlled period”. However, due to the fact that the completion rate *I _t_* at this stage is still relatively low, the number of active cases will continue to increase. The controlled period will continue until the second turning point *T*_2_ appears, that is, active cases *N _t_* reaches the peak and starts to decline. This is because the completion rate increase so significantly that *K _t_* = *I _t_* is fulfilled after a long period of treatment in the previous stage. When the completion rate *I _t_* surpasses infection rate *K _t_*, the number of patients treated in the hospital begins to decline from peak.

**Stage 3: Mitigation Period**

The sign of the end of the controlled period is *K _t_* = *I _t_*. Thereafter, *K _t_* will continue to fall with the rise of *I _t_*, which gives

$$K_{t}<I_{t},R_{t}=1+K_{t}-I_{t}>1\Rightarrow N_{t}<N_{t-1}$$

This indicates that the daily completion rate *I _t_* will start to be greater than the daily infection rate *K _t_*, that is, the value of the outbreak status indicator *R _t_* becomes less than 1. The population size with infectious capacity will be reduced, and the pressure of medical resources will be significantly relieved, marking the beginning of the “mitigation period”. The mitigation period will continue until the appearance of zero reported newly confirmed cases, that is, *E _t_* = 0, which we call the first “zero” point *Z*_1_. After the first “zero” point is reached, the intensity of prevention and control in the entire society will be relieved except for hospitals, that is, the “mitigation period” ends and the “convergence period” starts.

**Stage 4: Convergence Period**

The “convergence period” will end at the second “zero” point *Z*_2_, which means that the number of people treated in the hospital is equal to or close to “zero”. After reaching the second “zero” point, the epidemic is completely over.

For clarity, we summarize the iconic features and the corresponding milepost moments of each stage in the whole process of the epidemic in Table 1.

**Table 1.** The four stages of an epidemic.

Stage	Outbreak	Controlled	Mitigation	Convergence
Begin with	the number of newly diagnosed increases	the number of newly diagnosed decreases (the first turning point)	the number of patients in hospital decreases (the second turning point)	the number of newly diagnosed equals to 0 (the first “zero” point)
End with	the number of newly diagnosed reaches peak (the first turning point)	the number of active cases reaches peak (the second turning point)	the number of active cases equals to 0 (the first “zero” point)	the number of active cases equals to 0 (the second “zero” point)
	*K* ≫ I , *R* ≫ 1	*K* > I , *R* > 1	*K* < I , *R* < 1	*K* = 0 , *R* ≪ 1

### 2.3 Implementation: the proposed model

According to Section 2.2, the modeling and predicting of the epidemic need to be divided into two parts. The first part corresponds to the outbreak period, where the intervention and disease curing is not effective enough. The infection rate *K _t_* increases rapidly and the completion rate *I _t_* is small. Thus, the number of newly diagnosed patients *E _t_* increases rapidly, and the number of active cases *N _t_* increases. The pressure on medical resources will soon be overwhelmed. According to Equation (2), *N _t_* will be in an exponential growth trend without forming a convex curve, nor will the so-called two turning points or two “zero” points appear.

The second part, which is the focus of this article, is when the *K _t_* starts to decrease and *I _t_* starts to increase due to effective intervention and improved recovery level for individual patients. Only in this situation will the turning points and “zero” points *T*_1_, *T*_2_, *Z*_1_, *Z*_2_ successively appear, and then the epidemic could end. Therefore, we will model the development of the epidemic under the assumption of effective intervention, then we can obtain the early prediction of two turning points and two “zero” points based on the predicting modeling of *E _t_* and *N _t_*.

Suppose that the infection rate *K _t_* and the removed rate *I _t_* change gently with a stable unitary rate of change within a time window *m* before time *t*_0_, then given *m* and *t*_0_, denote *V*_*K*|( *t*_0_, *m*)_ and *V*_*I*|( *t*_0_, *m*)_ as the unitary rate of change of *K _t_* and *I _t_* respectively, that is,

$$V_{K|(t_{0},m)}={\left\{\frac{K_{t_{0}}}{K_{t_{0}-m+1}}\right\}}^{1/\left(m-1\right)},\;V_{I|(t_{0},m)}={\left\{\frac{I_{t_{0}}}{I_{t_{0}-m+1}}\right\}}^{1/\left(m-1\right)}.\;(3)$$

For any *t* > *t*_0_, the infection rate *K _t_* and the removed rate *I _t_* can be predicted as follows:

$${\hat{K}}_{t|t_{0}}:\text{=}{\hat{K}}_{t_{0}}(t-t_{0})={\hat{K}}_{t_{0}}(t-t_{0}-1)\cdot V_{K|(t_{0},m)}=\cdots ={\hat{K}}_{t_{0}}(1)\cdot V_{K|(t_{0},m)}^{t-t_{0}-1}=K_{t_{0}}\cdot V_{K|(t_{0},m)}^{t-t_{0}},\;(4)$$

$${\hat{I}}_{t|t_{0}}:\text{=}{\hat{I}}_{t_{0}}(t-t_{0})={\hat{I}}_{t_{0}}(t-t_{0}-1)\cdot V_{I|(t_{0},m)}=\cdots ={\hat{I}}_{t_{0}}(1)\cdot V_{I|(t_{0},m)}^{t-t_{0}-1}=I_{t_{0}}\cdot V_{I|(t_{0},m)}^{t-t_{0}}.\;(5)$$

Thus, we can obtain the outbreak status *R _t_*, the number of patients in the hospital *N _t_*, and the number of newly diagnosed *E _t_* as

$$\begin{array}{ccc} {\hat{R}}_{t|t_{0}} & = & 1+{\hat{K}}_{t|t_{0}}-{\hat{I}}_{t|t_{0}},\\ {\hat{N}}_{t|t_{0}} & = & {\hat{N}}_{t-1|t_{0}}\cdot {\hat{R}}_{t|t_{0}},\\ {\hat{E}}_{t|t_{0}} & = & {\hat{N}}_{t-1|t_{0}}\cdot {\hat{K}}_{t|t_{0}}. \end{array}$$

According to the prediction process, it can be seen that the prediction results mainly depend on *V*_*K*|( *t*_0_, *m*)_ and *V*_*I*|( *t*_0_, *m*)_, whose value is up to the selection of time window *m* and starting point *t*_0_. However, it is worth noting that the selection of *m* and *t*_0_ is not arbitrary, which is suggested as in the follow assumption.

**Assumption 1**. *The time window m and the starting point t*_0_ *should be chosen satisfy* *V*_*K*|( *t*_0_, *m*)_ < 1 *and* *V*_*I*|( *t*_0_, *m*)_ > 1. *Meanwhile, keeping I _t_* < 1 *due to interpretability constraints, and the starting point t*_0_ *should be close to the date of the latest published data as much as possible.*

It is worth noticing that the assumption is proposed to make sure that the trend of outbreak development have already emerged and stable, which means that the outbreak have already been controlled. The assumption is an mild requirement, since when some basic condition are satisfied, such as the epidemic prevention policy is effective and steady, the unitary rate of change would be relatively stable. Our method is totally based on the assumption above, thus when any constraint listed above is not satisfied, our algorithm would be inapplicable.

In summary, here we describe details of the proposed procedure in Algorithm 1.

**Algorithm 1. Main Prediction Procedure**

1: Initial setting *m* and *t*_0_, which satisfying Assumption 1;

2: Compute *V _K_* and *V _I_* according to ( 3); Set *t* = *t*_0_ + 1.

3: Prediction: updating the predicted results at time *t* via the forecasting value ahead of *l* = *t*− *t*_0_-step as follows:

$$\begin{array}{lll} {\hat{K}}_{t|t_{0}} & = & {\hat{K}}_{t_{0}}(l)=K_{t_{0}}\cdot V_{K|(t_{0},m)}^{l}\\ {\hat{I}}_{t|t_{0}} & = & {\hat{I}}_{t_{0}}(l)=I_{t_{0}}\cdot V_{I|(t_{0},m)}^{l}\\ {\hat{R}}_{t|t_{0}} & = & 1+{\hat{K}}_{t|t_{0}}-{\hat{I}}_{t|t_{0}}\\ {\hat{N}}_{t|t_{0}} & = & {\hat{N}}_{t-1|t_{0}}\cdot {\hat{R}}_{t|t_{0}}\\ {\hat{E}}_{t|t_{0}} & = & {\hat{N}}_{t-1|t_{0}}\cdot {\hat{K}}_{t|t_{0}} \end{array}$$

4: Prediction of the milepost moments: If *Ê*_*t*−1| *t*_0__ < *Ê*_*t|t _c_*_, then *T*_1_ = *t* − 1; If ${\hat{N}}_{t-1|t_{0}}<{\hat{N}}_{t|t_{0}},$ then *T*_2_ = *t* − 1; If *Ê*_*t*−1| *t*_0__ < *E*_0_ = 1, then *Z*_1_ = *t* − 1; If ${\hat{N}}_{t-1|t_{0}}<N_{0}=1,$ then *Z*_2_ = *t* − 1; If none of the above is satisfied, turn to the next step.

5: Set *t* = *t* + 1, return to Step 2 until *T*_1_, *T*_2_, *Z*_1_, *Z*_2_ are obtained.

It is also worth noting that in practice, there are many special cases that we need to take into consideration, thus we created a relatively complete computing framework, which has already been implemented and made into R packages and are available from **Github** (See data availability for more detail ( YuanchenZhu2020, 2020).

the more data we accumulate, the clearer the underlying law of the epidemic. Therefore, we can also continuously modify the iterative prediction model according to the actual data, so that the prediction of the next stage and the prediction of the long-term situation can be more accurate.

## 3 Application: Analysis of the COVID-19 in mainland China beyond Hubei Province

We apply our model to analyze and evaluate the COVID-19 using publicly available data from mainland China beyond Hubei Province from the China CDC during the period of Jan 29th, 2020, to Feb 29th, 2020. Here we first show the actual trend of the COVID-19, and then compared with the predicted ones via the proposed method. Finally, we will show the effect of *m* on the predicted results. All these results are implemented via **R** software.

### 3.1 The turning points and “zero” points observed

**Figure 1.**
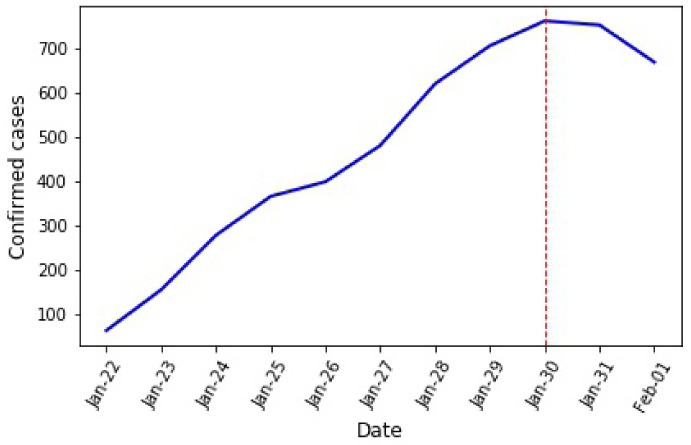
Trend of the daily confirmed cases from 01/22 to 02/01, 2020.

After the shutdown of most parts of Hubei province on Jan 23rd, other parts of China also immediately launched prevention and control strategies, including regional isolation, admission of all confirmed patients, isolating all suspected patients and so on. The effective implementation of these intervention policies quickly controlled the rapid spread of the epidemic in these areas. As can be seen in Figure 1, the parameter infectious rate *K _t_*, which reflects the intensity of the spread of the epidemic, has shown a significant downward trend since Jan 27th after severe fluctuations from Jan 22nd to 26th. As can be seen in Figure 1, we find out that the daily confirmed cases peaked on Jan 30th, 2020, with 761 confirmed cases and then continued to decline for two consecutive days.

However, the migration raised from people returning to work after Chinese New Year on Feb 3rd undermines the continuous decline of *E _t_*. Since Feb 2nd, the number of daily confirmed patients in mainland China beyond Hubei Province has increased for two consecutive days, where the *E _t_* on Feb 3rd has increased by 23% compared to that on Feb 2nd. It can be concluded that these fluctuations are caused by the resuming of social activities, which leads *E _t_* to continue to decline since Feb 4th. In many literature and media reports, Feb 3rd is used as the time point when the number of newly confirmed patients starts to decline. But considering the fact that the epidemic was already under control, here we still view Jan 30th as the first turning point.

**Figure 2.**
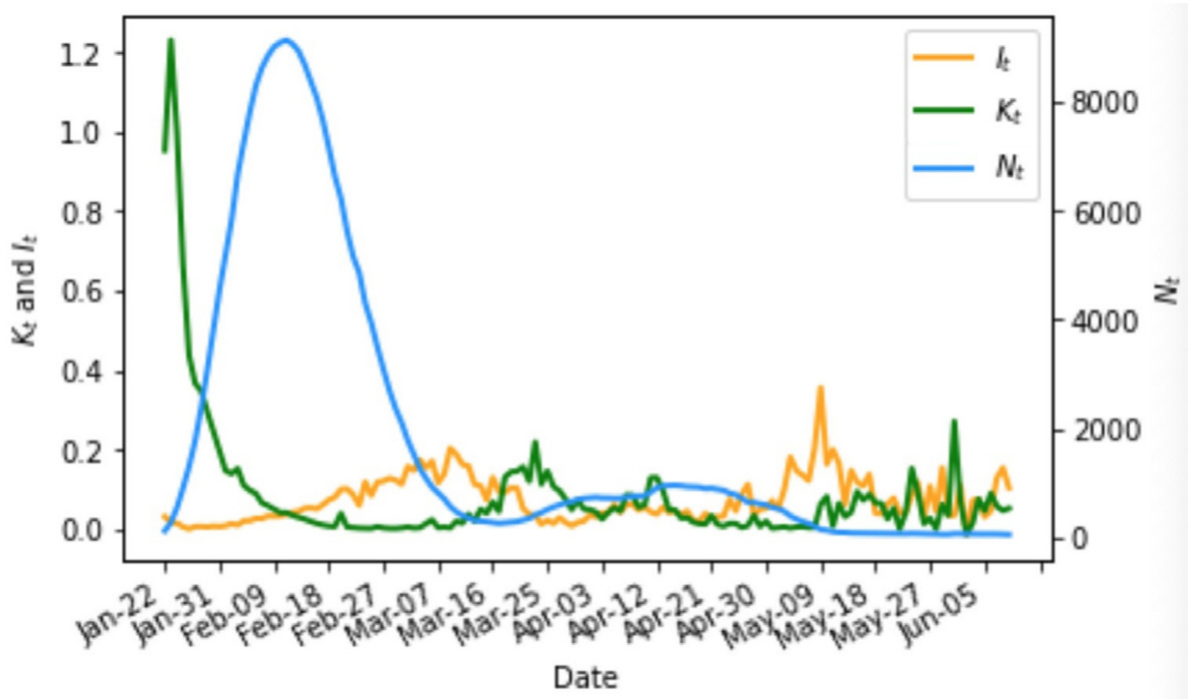
Observed *K _t_*, *I _t_* and *N _t_* of the COVID-19 from Jan 22 to Jun 10, 2020.

After that, the second turning point *T*_2_, which is the time point when the number of active cases *N _t_* starts to decline, is also observed. Figure 2 shows the true curves of the daily infection rate *K _t_*, daily removed rate *I _t_*, and *N _t_* calculated based on the actual data from mainland China beyond Hubei from Jan 22th, 2020 to Mar 13th, 2020. It can be seen that the second turning point *T*_2_ appeared on Feb 11th, with the emergence of *K _t_* < *I _t_* on that day, and the number of patients in the hospital continued to decrease since then.

As for the first “zero” point *Z*_1_, the definition is the time when the number of daily confirmed cases is equal to “zero”, which is too strict for the real situation. Thus, in this article, we take the criteria for cancelling travel warnings developed by the WTO during SARS as a reference, and make some adjustments to the definition of the first “zero” point: the time when the daily confirmed cases *E _t_* continues to be less than 5 for 3 days is revised to be *Z*_1_. Then, if we exclude confirmed cases that originated from abroad, daily confirmed cases has already become less than 5 since Mar 3rd in mainland China beyond Hubei Province, thus according to our revised definition, Mar 5th is *Z*_1_. However, there were still 1,089 active cases on that day. Therefore, it would still take some extra time to reach the second “zero” point *Z*_2_.

### 3.2 Prediction results

Starting from Jan 29th, we use the proposed forecasting method to make real-time predictions on the two turning points *T*_1_ and *T*_2_ and two ”zero” points *Z*_1_ and *Z*_2_ with window size *m* = 5. To clarify, the data before January 26th fluctuates violently, with assumption unsatisfied. Only after January 27 ^th^ the data becomes stable, thus we waited 2 days to make sure the trend had emerged and began our prediction at January 29 ^th^. The specific and predicted results are as follows.

We first conducted the proposed prediction model on Jan 29 ^th^, which indicated that the first turning point *T*_1_ would arrive on Jan 31st, i.e., *E _t_* < *E _t_* − 1. In reality, the first turning point did arrive on Jan 30th, which is only one day away from our predicted result.

**Figure 3.**
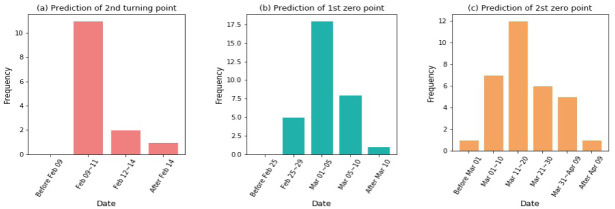
The frequency of prediction results of turning points and ”zero” points.

As for the second turning point, since the true *T*_2_ occurred on Feb 11th, we summarize the frequency of the prediction results obtained with *t*_0_ varying from Jan 29th to Feb 10th, 2020 and *m* = 5 in Figure 3(a). From it we can see that the prediction of the second turning point mainly concentrated in the range from Feb 9th to Feb 11th, which is consistent with the observed second turning point in reality. It is worth mentioning that we got the general information of *T*_2_ at a very early stage: we predicted on Feb 2nd that the second turning point *T*_2_ would arrive on Feb 11th, which is exactly the same as the second turning point that observed in reality. Since then, we have continuously tracked the rolling predictions, which have not yet changed much.

Similarly, Figure 3(b) and Figure 3(c) show the frequency of the prediction results for two “zero” points obtained with *t*_0_ varying from Jan 29th to Feb 29th, 2020 and *m* = 5, respectively. Specifically, for the predicted first “zero” point *Z*_1_ in Figure 3(b), we divide the prediction results from these days into 5 intervals, which can be seen that the prediction results of the first “zero” point *Z*_1_ are mainly concentrated on Mar 1st to 5th, which is consistent with the actual result. There is also a “pessimistic” prediction as a result of the sudden fluctuation of data on Feb 3rd, which predicted that the first “zero” point would arrive on Mar 17th. For the predicted second “zero” point *Z*_2_ in Figure 3(c), it can be seen that the second “zero” point will be reached from early-March to late-March. However, there is a prediction result that *Z*_2_ will appear on May 11th, which is far away from other results. The reason for this uncommon result is that the starting point of this forecast is Jan 29th, when the epidemic situation in mainland China beyond Hubei was still in the outbreak period with *E _t_* still rising, *I _t_* very small, so the prediction result about the finish of the epidemic may not be accurate.

**Figure 4.**
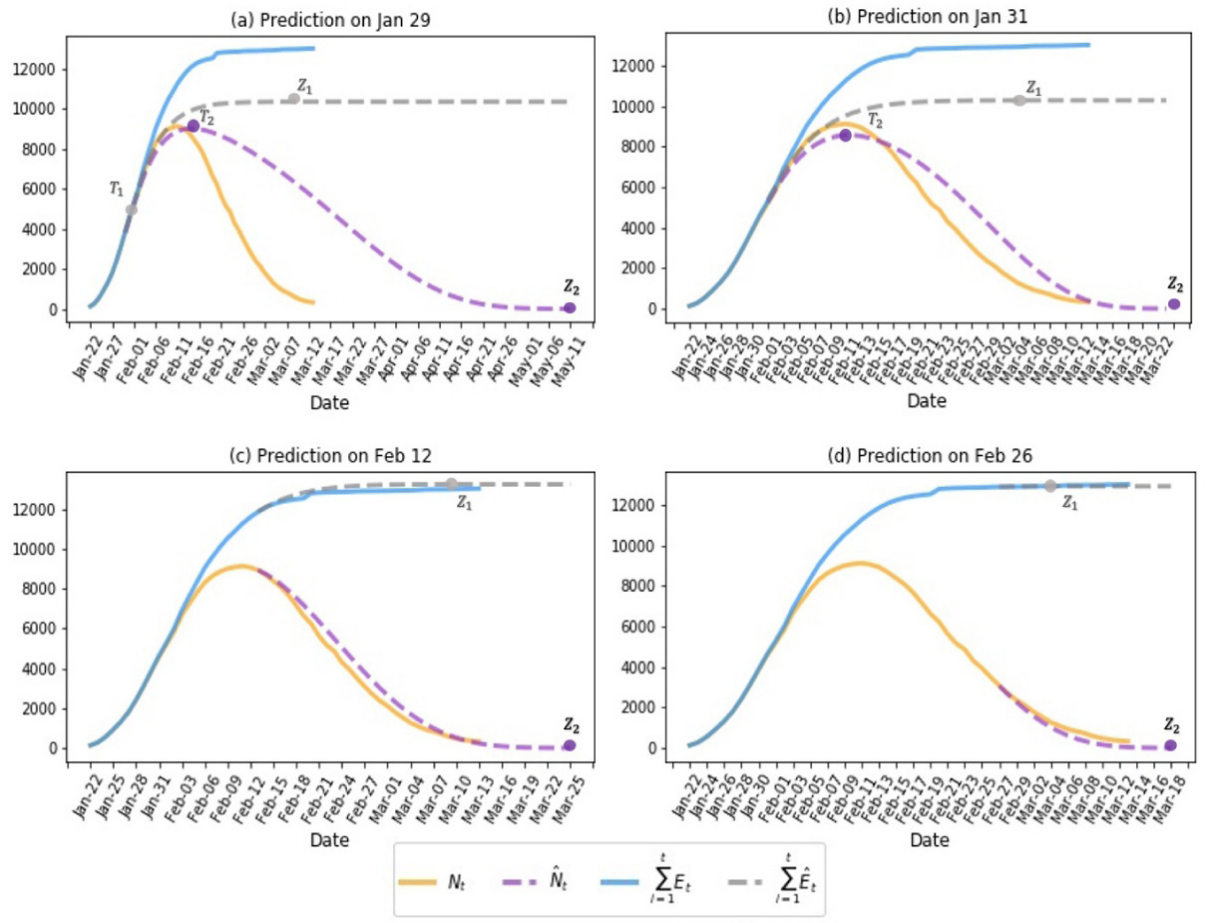
Forecasting results of the four milepost moments together with the trend of the cumulative number of active case ${\hat{N}}_{t}$ and the cumulative number of infectious $\sum_{l=1}^{t}{\hat{E}}_{l}$ compared with their observed cases *N _t_* and $\sum_{l=1}^{t}E_{l}$ when the prediction starting point *t*_0_ fixed at Jan 29th ( **a**), Jan 31st ( **b**), Feb 12th ( **c**) and Feb 26 ( **d**), 2020, respectively.

Furthermore, we also present the forecast results of the four milepost moments together with the trend of the cumulative number of active cases ${\hat{N}}_{t}$ and the cumulative number of infectious $\sum_{l=1}^{t}{\hat{E}}_{l}$ in Figure 4 when the prediction starting point *t*_0_ fixed at Jan 29th, Jan 31st, Feb 12th and Feb 26th, 2020, respectively. As can be seen from Figure 4(a), on Jan 29th, which is the very early stage of the epidemic, we predicted that the first turning point would appear on Jan 31st, which is only one day behind the actual observation. Additionally, the time of the second turning point result predicted on that day was Feb 14th, which is only 3 days away from the reality. The first ”zero” and second ”zero” forecast results are Mar 7th and May 11th, respectively.

Figure 4(b) shows the prediction results when the first turning point have already appeared, from which we can see that the prediction for *T*_2_ on Jan 31st is accurately with the second turning point possible occurring on Feb 11th. Meanwhile the first “zero” point and the second “zero” point are predicted to appear around Mar 4th and Mar 23rd, respectively.

Similarly, after the arrival of the second “zero” point, Figure 4(c) shows the forecast results of the first and second “zero” points predicted on Feb 12th, which show the forecast results for *Z*_1_ and *Z*_2_ are on Mar 9th and Mar 25th, respectively. From the fitting results, we know that our prediction of the cumulative number of active cases *N _t_* and the total number of confirmed patients is very similar to the actual situation, so our prediction results are likely reliable. Finally, we also give a very recent (Feb 26th) forecast in Figure 4(d), which is similar to the results mentioned above.

### 3.3 Results with different window sizes *m*

**Figure 5.**
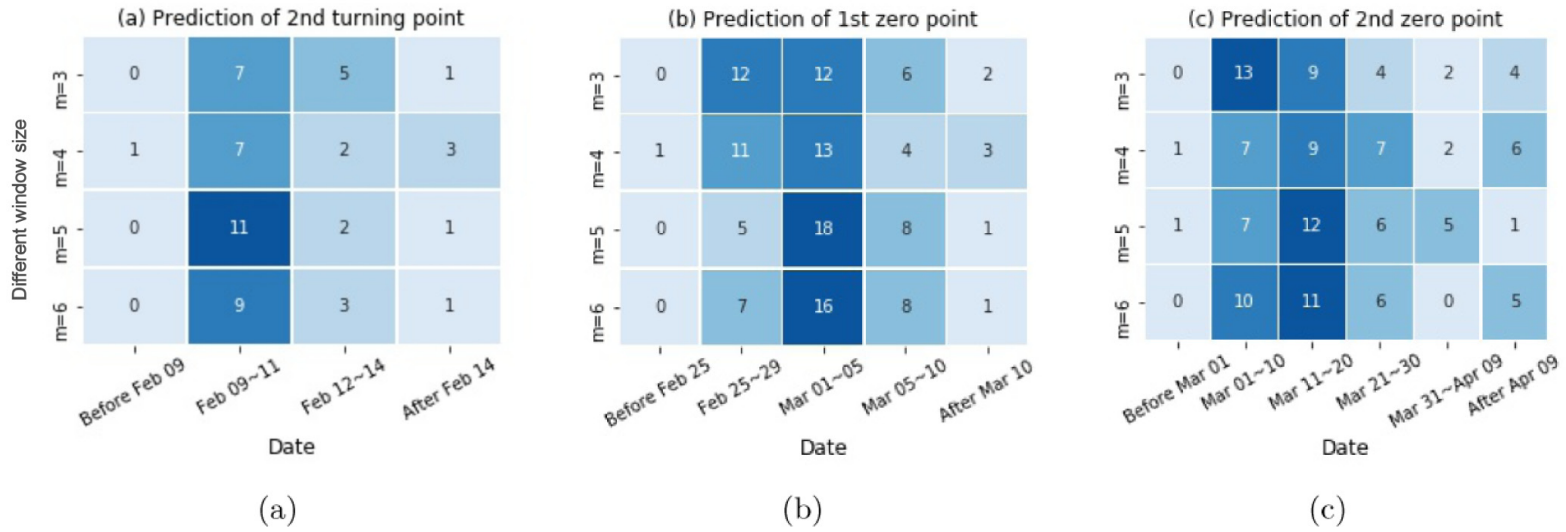
Summary of prediction for the second turning point ( **a**), the first ( **b**) and second ( **c**) “zero” points with different *m*.

Note that the number of *m* plays an important role in the proposed procedure, and all the results we discussed in the section 3.2 are obtained with fixed *m* = 5. In this section, we will illustrate the impact of different choice of *m* on the results, and give the empirical choice in real data analysis. Parallel to Section 3.2, here we obtain the results for the second turning point and both “zero” points via implementation of the proposed procedure with *m* =3, 4, and 6, respectively. We summarize all these results for the second turning point and both “zero” points in Figure 5, respectively.

From Figure 5, we can see that the highest frequency of prediction results for the second turning point occur around the period from Feb 9th to 11th for all choice of *m*, which means that the second turning point is most likely to occur during this period; similar results hold for the forecast of the first “zero” with the most likelihood of appearance around the early March. Both results show the limited influence of *m* on the results. From Figure 5(c), although the results of forecast frequency distributions for the second “zero” point with different *m* seem not as concentrated as those for the second turning point and the first “zero”, it varies slightly, with its occurrence from mid-March to mid-April. Overall, the choice of *m* seems not to be a critical value for the forecasting results, and we recommend its empirical choice from 3 to 6.

## 4 Discussion and conclusion

Focusing on the four meaningful mileposts, we put forward a simple and effective framework incorporating the effectiveness of the government control to forecast the whole process of a new unknown infectious disease in its early-outbreak. Specifically, we first propose a series of iconic indicators to characterize the extent of epidemic spread, and describe four periods of the whole process corresponding to the four meaningful milepost moments: two turning points and two “zero” points; then we develop the proposed procedure with mild and reasonable assumption, especially without relying on an assumption of epidemiological parameters for disease progression.

We examine our model with COVID-19 data in mainland China beyond Hubei province, which can detect the gross process of the epidemic at its early-outbreak. Specifically, in the first predicting task that conducted on Jan 29, the predicted date when the number of newly confirmed patients *E _t_* would fall for the first time is only one day behind the observation in reality. On Feb 2nd, our model predicted that the date when the number of patients in the hospital *N _t_* reaches its peak is Feb 11th, which is consistent with the real world situation. Later, the forecasting results fluctuated but were overall stable and close to the true observation. Meanwhile, we predict that the first “zero” point *Z*_1_ will arrive between the end of Feb and the beginning of March. And the second “zero” point *Z*_2_ will arrive at mid-March to mid-April. We also checked the robustness of our model under different time windows and found that the selection of the time window has little effect on the prediction of turning points. As a prediction model for the task of early warning of a new epidemic, our prediction model is proved to be quite efficient.

At present, many countries around the world are overwhelmed by the COVID-19 epidemic, which calls for global efforts. While our method is able to depict and predict the trend of an epidemic at a very early stage, it can be used to predict the current COVID-19 epidemic internationally, or any other new, unknown, explosive epidemic in the future. We believe that the prediction results of this method can provide decision support for epidemic control and intervention. It is worth noting that, due to the short-term dependence of our method, our model may show poor performance for wildly fluctuating data. Thus, more data preprocessing methods like data smoothing need to be developed within our framework, in order to allow for wider use of our method.

## Data availability

The underlying data and code required to replicate the studies finding are available from GitHub (data: https://github.com/Vicky-Zh/Tracking_and_forecasting_milepost_moments_of_COVID-19/tree/v1.0.0, code: https://github.com/YuanchenZhu2020/DemoPreTurningPointsCOVID19) and archived with Zenodo (data: http://doi.org/10.5281/zenodo.3755197 ( Zhang, 2020), code: https://doi.org/10.5281/zenodo.3987242 ( YuanchenZhu2020, 2020)).

### Underlying data

Zenodo: Vicky-Zh/Tracking and forecasting milepost moments of COVID-19: First release. http://doi.org/10.5281/zenodo.3755197 ( Zhang, 2020).

This project contains the following underlying data:

- Data of China Mainland Beyond Hubei.csv (A csv file with data collected from China CDC and four variables: the cumulative confirmed cases up to the given day *t*, the daily confirmed cases at day *t*, the daily recovered ones and the daily deaths at day *t*, with *t* from Jan 29th to Feb 29th, 2020)

### Extended data

Zenodo:

YuanchenZhu2020/DemoPreTurningPointsCOVID19:

Version 1.0.0. https://doi.org/10.5281/zenodo.3987242 ( YuanchenZhu2020, 2020).

This project contains the following extended data:

- **DemoPreTurningPointsCOVID19_1.0.0.zip** (R binary package)
- **DemoPreTurningPointsCOVID19_1.0.0.tar.gz** (R source package)
- **DemoPreTurningPointsCOVID19_1.0.0.pdf** (Reference manual for R package)

Data are available under the terms of the Creative Commons Zero ”No rights reserved” data waiver (CC0 1.0 Public domain dedication).
