# Supplementary material for: Tracking and forecasting milepost moments of the epidemic in the early-outbreak: framework and applications to the COVID-19
Source: F1000Res. 2020 Sep 18;9:333. Originally published 2020 May 6. [Version 2] doi: 10.12688/f1000research.23107.2 (PMC7737706; doi:10.12688/f1000research.23107.2)
Supplement: Peer review history

# Peer review: Reviewer response for version 2

**DOI:** 10.5256/f1000research.28903.r71640

**Published:** 2020-12-14

**Authors:** Paula Brito

**Affiliations:** 1 Faculty of Economics, University of Porto & LIAAD - INESC TEC, Porto, Portugal

In this new version, the authors have clarified the issues raised, mainly about the applicability of the proposed method, and the choice of the starting point.

 As mentioned before, the results reported from the application of the proposed methodology to the China Covid-19 data are quite good, showing that the method is well founded and useful.

 I look forward to seeing this being applied to data from other regions/countries, and results discussed.

 The manuscript is quite well written, and is easy to follow. There are nevertheless some typos/small issues that need correction, as indicated here-below.

 I consider that the method is sound, and not complicated, neither relying on extraordinary assumptions.

 I consider this is an interesting and nice piece of work, in a pertinent and up-to-date topic, and I am therefore in favour of acceptance.

**Minor issues :**

 Page 3, line 3 : reported in -> reported on

 Page 3, line 5 : stopping the spread -> stopping of the spread

 Page 3, line 12 : of highly -> highly

 Page 3, line 32 : is as high -> as high

 Page 4, line 26 : correct “specifically”

 Page 4, line 38 : proposed  -> propose

 Page 4, line 39 : defined  -> define

 Page 4, line 42 : applied  -> apply

 Page 5, line 20, formula for N _t_ : in the sum, is it E _t_ or E _i_ ?

 Page 5, last line : the right side of the formula should be:  N _0_ (1 + K –I ) ^t^   (there is a ‘1’ instead of ‘I’ )

 Page 6, line 22 : data  -> date

 Page 6, line (-13) : K _t_ < I _t_ ,R _t_ =1+ K _t_ − I _t_ < 1⇒ N _t_ < N _t__−1_  - in the center it should be ‘<’ and not ‘>’, R _t_ < 1

 Page 6, line (-10) : pressure of -> pressure on

 Page 6, lines (-4)-(-3) : number of people treated in the hospital -> number of active cases

 Page 7, Table 1, cell in line 1, column 3 : patients in hospital  -> active cases

 Page 7, line (-11) : number of patients in the hospital -> number of active cases

 Page 7, line (-3) :  chosen satisfy -> chosen to satisfy

 Page 8, lines 1-2 :  have already emerged and stable -> has already emerged and is stable

 Page 8, line 2 :  outbreak have already -> outbreak has already

 Page 8, line 3 :  are satisfied  ->  is satisfied

 Page 8, Algorithm 1, point 1 : remove “which”

 Page 8, line 5 after the Algorithm box :  the -> The

 Page 8, line (-19) :  compared -> compare

 Page 8, line (-13) :  Figure 1 -> Figure 2

 Page 9, line 3 : number of patients in the hospital -> number of active cases

 Page 9, lines 13 and 14 : “the data”: please indicate what you refer to exactly

 Page 10, line 1 : Please correct : *E _t_*< *E _t _*_−1   _(index of the second term)

 Page 10, line 9 : that -> that was

 Page 10, line (-4) : “after the arrival of the second “zero” point, Figure 4(c) shows…” - is it “zero point” or “turning point” here ?...

 Page 11, line 4 : number -> value

 Page 11, line 5 : section -> Section

 Page 11, line (-10) : choice -> choices

 Page 12, line 4 : assumption -> assumptions

 Page 12, line 6 :  that conducted ->  that was conducted.

Is the work clearly and accurately presented and does it cite the current literature?

Yes

If applicable, is the statistical analysis and its interpretation appropriate?

Yes

Are all the source data underlying the results available to ensure full reproducibility?

Yes

Is the study design appropriate and is the work technically sound?

Yes

Are the conclusions drawn adequately supported by the results?

Partly

Are sufficient details of methods and analysis provided to allow replication by others?

Yes

Reviewer Expertise:

Multivariate Data Analysis

I confirm that I have read this submission and believe that I have an appropriate level of expertise to confirm that it is of an acceptable scientific standard.

# Peer review: Reviewer response for version 2

**DOI:** 10.5256/f1000research.28903.r71641

**Published:** 2020-10-09

**Authors:** Rosanna Verde

**Affiliations:** 1 Department of Mathematics and Physics, University of Campania “Luigi Vanvitelli”, Caserta, Italy

I completely agree with the new version.

 The authors have improved the paper also following the comments and suggestions I gave.

 The paper is interesting and the proposed methods could be applied to COVID-19 data of other Countries.

Is the work clearly and accurately presented and does it cite the current literature?

Yes

If applicable, is the statistical analysis and its interpretation appropriate?

Yes

Are all the source data underlying the results available to ensure full reproducibility?

Yes

Is the study design appropriate and is the work technically sound?

Yes

Are the conclusions drawn adequately supported by the results?

Yes

Are sufficient details of methods and analysis provided to allow replication by others?

Yes

Reviewer Expertise:

Statistical Data Analysis; Data Stream Analysis; Functional Data Analysis; Symbolic Data Analysis; Clustering

I confirm that I have read this submission and believe that I have an appropriate level of expertise to confirm that it is of an acceptable scientific standard.

# Peer review: Reviewer response for version 1

**DOI:** 10.5256/f1000research.25510.r63123

**Published:** 2020-07-14

**Authors:** Paula Brito

**Affiliations:** 1 Faculty of Economics, University of Porto & LIAAD - INESC TEC, Porto, Portugal

In this paper, the authors propose a methodology aimed at forecasting milepost moments of an epidemic in the early outbreak. This is then applied to the COVID-19 outbreak in China.

 The method lays on the definition of the relevant involved variables :

- E _t_ : number of new confirmed cases at date t
- O _t_ : number of recovered cases at date t
- D _t_ : number of deaths at date t
- N _t_ : number of infectious cases in hospital at date t
- K _t_ : infection rate at date t
- I _t_ : removal rate at date t
- R _t_ : outbreak status at date t

The milepost moments to be predicted are

- the first turning point, T1 – when the number of newly diagnosed patients E _t_ starts decreasing;
- the second turning point, T2 – when number of active cases N _t_ starts decreasing;
- the first zero point, Z1– when the number of newly diagnosed patients E _t_ becomes null ;
- the second zero point, Z2– when the number of active cases N _t_ becomes null.

The procedure depends on the starting point t _0_, and on the size of the time window used, m. It is assumed that K _t_ and I _t_ change gently within time window m before t _0_, so that the average change rate of both K _t_ and I _t_ within that period are reliable values.

 Under those assumptions, recurrence formulas allow predicting the required time points.

 The results reported from the application of the proposed methodology to the China Covid-19 data are quite good, predicting well the observed time points, and hence show that the method is well founded and useful.

 The manuscript is quite well written, and is easy to follow.

 The pertinence and actuality of the topic go without saying. The method appears sound to me, and is not extremely complicated, neither relying on extraordinary assumptions.

 I consider this is an interesting and nice piece of work, and I am therefore in favour of acceptance.

 However, a few issues need consideration/correction, as described here below.

 The authors should revise the manuscript taking these aspects into account.

**Major points**

1. The success of the proposed methodology lays on the fact that the values of the average change rate of both K _t_ and I _t_, within the considered time window period, are reliable and stable - so that they may be used for prediction. It is not clear to me how this condition may be assessed, or what are the criteria involved. This should be clarified.
  The fact that the reported predictions for China are indeed good, result, in my opinion, from the fact that the local situation has been stable, measures taken and local conditions not changing before the general end of the outbreak (or so it is perceived from the outside). If this were not the case, I wonder whether the method would still apply – which then questions its applicability in other countries /regions where such stability is not guaranteed/observed.
  This emphasises the need for a clear establishment of applicability conditions.
  This is my major concern.
2. I am curious about the prediction results if the starting point is a bit earlier, and not as close to T1 as January 29 ^th^.
3. N _t_ is defined as “number of infectious cases in hospital at date t”. I believe this is what is generally referred to as “active cases” – also because in many countries not all diseased people are actually in hospital. I suggest using this terminology, so that it becomes clear to a general reader. In that case, the reference to “in hospital” should be updated along the manuscript.
4. Algorithm 1 : there are some typos here, that require attentive correction: in point 3,
  - formula 1: it should be K and not R , in both left and middle terms
  - formula 3, right term: R should be replaced by K

 
**Minor issues**

 Page 1, line 15 of the Abstract : assumption -> assumptions

 Page 1, line 16 of the Abstract : COVID-19 -> COVID-19 outbreak

 Page 1, line 25 of the Abstract : counties -> countries

 Page 3, line 35 : results -> result

 Page 4, line 30 : generalized -> used

 Page 4, line 36 : assumption -> assumptions

 Page 8, Algorithm 1, line 2 : satisfying -> satisfy

 Page 8, Section 3, line 7 : in Jan 23rd -> on Jan 23rd

 Page 9, line 12 : decreases -> decrease

 Page 10, line 15 : in Jan 29th -> on Jan 29th

 Page 10, line (-13) : zero -> turning

 Page 11 – Figure 5 : the labels along the horizontal axis are not centered, therefore the figure is not clear.

Is the work clearly and accurately presented and does it cite the current literature?

Yes

If applicable, is the statistical analysis and its interpretation appropriate?

Yes

Are all the source data underlying the results available to ensure full reproducibility?

Yes

Is the study design appropriate and is the work technically sound?

Yes

Are the conclusions drawn adequately supported by the results?

Partly

Are sufficient details of methods and analysis provided to allow replication by others?

Yes

Reviewer Expertise:

Multivariate Data Analysis

I confirm that I have read this submission and believe that I have an appropriate level of expertise to confirm that it is of an acceptable scientific standard, however I have significant reservations, as outlined above.

# Peer review: Reviewer response for version 1

**DOI:** 10.5256/f1000research.25510.r63126

**Published:** 2020-06-17

**Authors:** Rosanna Verde

**Affiliations:** 1 Department of Mathematics and Physics, University of Campania “Luigi Vanvitelli”, Caserta, Italy

The paper deals with a forecasting procedure to analyse and evaluate the early stage of the COVID-19 outbreak in China. Inspite the classical SEIR model, the authors define a set of iconic indicators to study the status of disease contagion and the extent of epidemic spread. Then, they divide the cycle of the epidemic in four stages corresponding to the four meaningful milepost moments: two turning points and two “zero” points, related to the tuning of the proposed indicators.

 The authors characterize the first stage: the *Outbreak period*, by its end, which is represented by the arrival of the first tuning point (T_1), when the newly diagnosed patients (denoted E_t) after a rapid rise, begin to decrease (E_t < E_{t-1}). The second stage is the *Controlled period*, that sees an increasing of hospitalised patients (denoted N_t) until a second turning point (T_2), when N_t, after to have reached a peak, starts to decline.

 The *Mitigation stage* corresponds to the third stage and it continues until the daily confirmed cases reach the zero (E_t=0). That represents the first “zero” point (Z_1). This condition is also reformulated and referred to the time when the daily confirmed cases are less then “5” for 3 consecutive days.  

 The *Convergence Period*, the four stage, is characterized to reach a second “zero” point (Z_2), when the number of hospitalised patients is closed to zero (N_t=0). That signs the end of the epidemic.

 The procedure is corroborated on public available data in mainland China beyond Hubei Province from the China CDC during the period of Jan 29th, 2020, to Feb 29th, 2020. The results show the proposed procedure has provided to a prediction of the tuning and “zero” points, very near to the exact dates also in a very large stage of the epidemic.

 An analysis of the robustness of the method has also been performed on the time windows and on the four milepost moments. The results have revealed the influence of the parameter “m”, related to the width of the time windows used to estimate the average change rate of the daily diagnosed cases and of the daily removed cases.

 The paper is clear and smoothly written. I agree for its indexing after to have kept into consideration the following remarks:

 Section 2. Methods

 Equation (1)

 1) N_l in brackets (1+K_l - N_l) should to be replaced with I_l

 Section 2.3

 2) I suggest of referring explicitly the exponential model of the infection rate K_t and of the removed rate I_t (in the window m);

 3) the term “average” for V_{K|(t0,m)}  (and V_{I|(t0,m)}) is confused, it would be better to refer to it as “unitary rate of change” (and “unitary rate of removed”). I think, that is more properly the “rate of change” (and the “rate of removed”) associated with a unitary time step.

 Algorithm 1 Main Prediction Procedure.

 3 Prediction: updating the predicted results …..

 4) The first equation R_t|to = \hat{R_t|to (l)} should to be replaced with K_t|to = \hat{K_t|to (l)}

 Section 3: Application

 5) All the Figures must be improved, especially the Figure 2. And the Figure 4.

 6) The proposed strategy has been verified on data referred to a very early stage of the epidemic and it performs short-term dependency. Given to the spread of the epidemic in world wide, maybe it can be proved on data related to the outbreak of the pandemic in another Countries.

Is the work clearly and accurately presented and does it cite the current literature?

Yes

If applicable, is the statistical analysis and its interpretation appropriate?

Yes

Are all the source data underlying the results available to ensure full reproducibility?

Yes

Is the study design appropriate and is the work technically sound?

Yes

Are the conclusions drawn adequately supported by the results?

Yes

Are sufficient details of methods and analysis provided to allow replication by others?

Yes

Reviewer Expertise:

Statistical Data Analysis; Data Stream Analysis; Functional Data Analysis; Symbolic Data Analysis; Clustering

I confirm that I have read this submission and believe that I have an appropriate level of expertise to confirm that it is of an acceptable scientific standard, however I have significant reservations, as outlined above.
